# Impact of Red Spinach Extract Supplementation on Bench Press Performance, Muscle Oxygenation, and Cognitive Function in Resistance-Trained Males

**DOI:** 10.3390/sports9060077

**Published:** 2021-05-27

**Authors:** James T. Haynes IV, Jeremy R. Townsend, Marko A. Aziz, Megan D. Jones, Laurel A. Littlefield, Matthew D. Ruiz, Kent D. Johnson, Adam M. Gonzalez

**Affiliations:** 1 Exercise and Nutrition Science Graduate Program, Lipscomb University, Nashville, TN 37204, USA; jthaynes@mail.lipscomb.edu (J.T.H.IV); maaziz@mail.lipscomb.edu (M.A.A.); mdj016@uark.edu (M.D.J.); llittlefield1@lipscomb.edu (L.A.L.); mdruiz@lipscomb.edu (M.D.R.); kdjohnson@lipscomb.edu (K.D.J.); 2 Department of Health Professions, Hofstra University, Hempstead, NY 11549, USA; Adam.M.Gonzalez@hofstra.edu

**Keywords:** nitric oxide, resistance training, strength, power, nitrates

## Abstract

The purpose of this study was to assess the impact of short-term dietary nitrate supplementation, in the form of red spinach extract (RSE), on bench press performance, muscle oxygenation, and cognitive function in resistance-trained males. Ten resistance-trained males participated in this randomized, cross-over, placebo-controlled, double-blind investigation. Each participant completed 7 days of either RSE (2 g; 180 mg NO_3_^−^) or a maltodextrin placebo (PL) in a counterbalanced fashion with a 14-day washout between treatments. During experimental visits, participants were provided their 8th and last dose of RSE or PL 40 min before completing 5 sets of the barbell bench press exercise to failure at 75% of a predetermined 1-repetition maximum with 2 min rest intervals. Mean and peak power were recorded via a linear transducer. Near-infrared spectroscopy (NIRS) was implemented to estimate muscle oxygenation, a Stroop Test was used to assess cognitive function, and subjective performance ratings were obtained in relation to the acute resistance exercise sessions. Data were analyzed via separate repeated measures analyses of variance. There were no time by group interactions for bench press repetitions (*p* = 0.549), peak power (*p* = 0.061), or mean power (*p* = 0.877) across the 5 sets of bench press. Additionally, no significant differences (*p* > 0.05) were observed for any measure of muscle oxygenation, Stroop performance, or subjective performance ratings. It appears that 7 days of RSE supplementation did not alter performance, muscle oxygenation, nor Stroop scores during or following the bench press exercise in resistance-trained males.

## 1. Introduction

Nitric oxide (NO) is a molecule produced by the body which stimulates a variety of actions including vasodilation [1], improved calcium handling [2], improved exercise economy [3], and increased velocity of skeletal muscle contractions [4]. Dietary nitrates (NO_3_^−^) act as a precursor to NO and can be consumed through NO_3_^−^-rich vegetable products, most commonly beetroot juice (BRJ) [5]. Following consumption, NO_3_^−^ is converted to nitrite (NO_2_^−^) via anaerobic bacteria on the surface of the tongue and through the NO_3_^−^ -NO_2_^−^ pathway, NO_2_^−^ is eventually reduced to produce NO which then can elicit its biological effects on various cells in the body [6]. Through these mechanisms, dietary NO_3_^−^ supplementation has been shown to improve exercise performance by improving muscle’s ability to use oxygen [7], time-trial performance [8], and high-intensity intermittent running performance [9]. Though, not all studies have shown an ergogenic effect of BRJ on high-intensity exercise performance [10,11,12]. Furthermore, some data suggests that NO_3_^−^ -rich foods can enhance cerebral perfusion to areas of the brain responsible for executive functioning [13], which may enhance cognition during and following fatiguing exercise [14,15].

To date, the majority of research regarding dietary NO_3_^−^ and exercise performance has focused on endurance exercise with less attention given to resistance exercise. However, it has been suggested that resistance-trained athletes may benefit from NO_3_^−^ supplementation by increasing force output and reducing the fatigue of fast twitch muscle fibers via improved calcium handling [2]. Additional mechanisms by which NO_3_^−^ supplementation may contribute to improved resistance exercise performance include enhanced neuromuscular efficiency [16], a reduced adenosine triphosphate-phosphocreatine (ATP-PCr) cost of exercise [17], and increased blood flow. Specifically, type II muscle fibers may be preferentially affected by NO_3_^−^ due to their increased capability for NO_3_^−^ storage [18] and improved blood flow [19]. As insufficient O_2_ delivery to the active musculature is a limiting factor to ATP-PCr resynthesis [20], dietary NO_3_^−^ may allow for the maintenance of force production during resistance exercise.

A small number of studies have been published of late regarding resistance exercise and nitric oxide precursors. Initial work by Mosher et al. [21] revealed that a short loading phase (6 days) increased total volume lifted during bench press resistance exercise at 60% of 1 repetition maximum (1RM). Williams and colleagues [22] subsequently showed that acute BRJ supplementation increased mean velocity and mean power output across 3 sets of bench press to repetitions failure. However, data has been unclear with other work reporting improvements in number of repetitions performed at 60% and 70% 1RM only in the back squat and no improvements for the bench press exercise in healthy males [23]. Previous work from our lab indicated that acute administration of a concentrated high-NO_3_^−^ supplement significantly increased peak isometric force production in adolescent males but did not improve repeated sprint performance compared with a placebo (PL) [24]. Yet, results from Trexler et al. [25] demonstrated that BRJ provided no ergogenic effect during maximum leg extensions, nor did it improve femoral artery blood flow. Since research regarding NO_3_^−^ supplementation on resistance exercise to date is equivocal, more research is needed to elucidate the potential ergogenic effects. 

Red spinach extract (RSE) is a rich source of dietary NO_3_^−^ that increases plasma NO_3_^−^/NO_2_^−^ levels 30 min post consumption [26,27] and has recently been introduced to the consumer market as a new dietary NO_3_^−^ supplement. Moreover, a 1 g dose of RSE significantly increased ventilatory threshold during a graded exercise test commencing at 65–75 min post-ingestion compared to PL [28]. Regarding exercise performance, Gonzalez et al. [8] found that 7 days of RSE supplementation, in addition to consuming the supplement 60 min before exercise, significantly reduced time-to-completion and increased measures of power and speed during a 4 km cycling time trial. Although evidence supporting the consumption of dietary NO_3_^−^ to enhance resistance exercise seems positive, no studies have examined the effects RSE on acute resistance exercise performance. Additionally, prior work suggests that the ergogenic benefits of dietary NO_3_^−^ may be specific to the source of NO_3_^−^ [29], signifying the need for more data regarding various sources of dietary NO_3_^−^.

Therefore, the current study aimed to investigate the effects of 7 days of RSE supplementation on bench press performance, muscle oxygenation, and cognitive performance in resistance-trained males. We hypothesized that short-term RSE supplementation would improve muscle oxygenation, increase the number of total repetitions completed during a fatiguing bench press protocol, attenuate reductions in mean and peak power, as well as improve subjective measures of fatigue. Furthermore, we hypothesized that short-term RSE supplementation would attenuate reductions in cognitive performance following the fatiguing bench press protocol.

## 2. Methods

Participants reported to the laboratory on 3 separate occasions. During a baseline visit (visit 1), researchers collected anthropometric, body composition, and maximal strength measures as well as familiarized participants with various aspects of the experimental protocol (e.g., Stroop effect assessment). Participants were then randomly assigned to their first study treatment utilizing a double-blind, crossover, placebo-controlled design to receive either a RSE or PL treatment using a random number generator. Participants consumed 2 g of either RSE or PL daily, for an acute loading phase of 7 days, with an additional dose on the day of the testing visits. During visits 2 and 3, participants completed cardiovascular assessments, a series of cognitive Stroop tasks and visual analog scales (VAS), and a muscular fatiguing bench press protocol. A period of at least 14-days separated experimental trials for a sufficient washout period. All procedures were approved by the Lipscomb University Institutional Review Board and each participant provided his informed consent prior to enrollment. This trial was registered at clinicaltrials.gov (accessed on 12 April 2021) as NCT04292106.

### 2.1. Participants 

A total of 10 resistance-trained males (22.6 ± 3.2 years; 178.7 ± 7.4 cm; 88.3 ± 7.8 kg), with an average length of resistance training experience of 7.4 ± 3.1 years and a bench press 1RM of 108.4 ± 29.0 kg participated in this study. Using a power of 0.8 and a significance level of 0.05, it was determined that at least 10 participants were required to detect significance based on changes in repetitions to fatigue in previous work [22,30]. For each visit to the lab, participants were instructed to arrive following a 10 h fast, to avoid strenuous exercise for 72 h, and to abstain from alcohol and caffeine consumption for 24 h. All participants had recently been utilizing training protocols similar to what was prescribed in this study (3–5 sets, 6–12 repetitions,1–2 min rest). Each participant recorded their dietary intake for 3 days the week of supplementation consisting of one weekday, one weekend day, and the day before their testing visit. Following visit 2, participants were provided a copy of their dietary recall and instructed to follow it as closely as possible leading up to visit 3. The time of all trials were completed ± 2 h of the same time of day.

### 2.2. Anthropometrics and Body Composition Assessment

Height was recorded in centimeters and measured via stadiometer. Body mass, non-bone fat-free mass (FFM), and body fat percentage was determined using whole body-dual energy X-ray absorptiometry (DXA) scans (Prodigy^TM^; Lunar Corporation, Madison, WI, USA). Total body estimates of percent fat and non-bone FFM (±0.1 kg) was determined using company’s recommended procedures and supplied algorithms. Daily calibrations of quality assurance were completed prior to all DXA scans using the manufacturer supplied calibration block. All DXA assessments were completed using standardized subject positioning procedures by a single certified radiological technician.

### 2.3. One Repetition Maximum (1RM) Testing

During the baseline visit to the laboratory, each participant was tested for their bench press 1RM in order to determine the appropriate load during visits 2 and 3. Briefly, each participant performed a standardized series of dynamic exercises followed by 2 bench press warm-up sets using a resistance of approximately 40–60% of their estimated 1RM for 6–10 repetitions and 60–80% of estimated 1RM for 3–5 repetitions, respectively. Subsequently, the resistance load was increased conservatively over the course of 3–5 maximal trials (1-repetition sets) to determine the 1RM. Each maximal trial was separated by 3–5 min of rest. The 1RM was recorded as the maximum load that the participant could lift for 1 repetition while maintaining proper technique. Additionally, each participant’s hand placement on the barbell was measured and recorded for the bench press as a reference for the participant’s hand placement during the following visits. During visits 2 and 3, athletic tape was placed on the bar to indicate where the participant should place their hands and reduce variability with the grip the participants used.

### 2.4. Supplementation Protocol

Participants consumed 2 g of RSE or PL for 7 days leading up to the 2nd and 3rd testing visits with an additional dose 40 min before engaging in a warm-up to complete the resistance exercise protocol. Previous work has shown elevated plasma NO_3_^−^/ NO_2_^−^ levels 30 min post consumption [26,27] following RSE consumption and other work has utilized a similar pre-exercise wash-in period [8]. The supplement was consumed via capsule form with each dose consisting of 4 capsules. Each RSE capsule consisted of 500 mg RSE (Super Spinach; NuVital Health, Long Beach, NY, USA) whereas each PL capsule consisted of 500 mg maltodextrin. Participants were instructed to refrain from using antibacterial mouthwash during the supplementation period due to its potential to reduce NO_3_^−^ bioavailability by disrupting bacteria in the mouth required to convert NO_3_^−^ to NO_2_^−^ to NO [31]. Furthermore, participants were also asked to take the supplement during the middle of the day or at a different time of the day other than when they brushed their teeth, for the previously mentioned reason. Participants were also advised to take the supplement at the same time every day. 

### 2.5. Experimental Trials

Upon arrival to the laboratory, participants took a seat and answered a series of questions to confirm their compliance with the pre-visit instructions discussed earlier. Next, participants remained seated with legs uncrossed, while 2 recordings of their heart rate (HR) and blood pressure (BP) were obtained. Subsequently, participants completed their first VAS and Stroop test. Afterwards, participants consumed their last dose of either RSE or PL and underwent a rest period of 30 min to allow the NO_3_^−^ reduction process to occur. At 30 min post consumption, subjects completed a second Stroop test. A 30 min post consumption HR and BP were also recorded. Thereafter, participants completed a 5 min warm-up on a cycle ergometer and a short general dynamic warm-up followed by a bench press specific warm-up that consisted of: 10 repetitions with the bar, one set of 40% of their 1RM for 8 repetitions, and one set of 60% of their 1RM for 4 repetitions. After the warm-up was completed, subjects completed a second VAS. Upon arrival of the 1-h post ingestion time mark, participants completed 5 sets to fatigue at 75% of their 1RM on the bench press. Each set was separated by 2 min of rest while remaining seated. Participants were instructed to use proper form and complete as many repetitions as possible until failure. Failure was defined as the inability to complete a full repetition without assistance. Researchers recorded the total number of repetitions completed during each set. Immediately after completion of the bench press exercise, participants were moved to a seat to complete their third VAS scale and allow the researchers to obtain a final HR and BP assessments. Lastly, the participants completed a third Stroop test before leaving the laboratory.

#### 2.5.1. Heart Rate and Blood Pressure

For the two testing visits, participant HR and BP were recorded at 3 different time points throughout the study: upon arrival, 30 min post consumption and immediately after completion of the fatiguing bench press exercise. Measurements of HR and BP were obtained using an automatic blood pressure monitor (Model MDS4001, Medline industries, Inc., Mundelein, IL, USA). Each measurement of HR and BP were recorded twice and the average of the 2 measurements were used for analysis.

#### 2.5.2. Power Measures 

Power output during the barbell bench press exercise was measured for each repetition with a linear position transducer (Gym Aware, Mitchel, Australia). The linear position transducer attaches to the end of the barbell, which measures linear displacement and time to calculate mean and peak barbell velocity. Power was calculated from the barbell load entered into the microcomputer and barbell velocity detected by the unit. Peak power (PP) and mean power (MP) outputs were recorded for each repetition. For subsequent analysis, the average PP and MP output values were calculated for each set. The GymAware device has been previously demonstrated to show high reliability for the bench press exercise [32].

#### 2.5.3. Muscle Oxygen Saturation (SmO_2_) Assessment and Data Analysis

To provide an estimate of oxygen saturation in the active musculature, a near-infrared spectroscopy (NIRS) device (MOXY, Hutchinson, MO, USA) was affixed to the anterior deltoid similar to previous work utilizing NIRS technology to monitor the bench press exercise [33]. Specifically, the surface of the skin was prepped with an alcohol swap and excess hair was removed if necessary. The NIRS device was then centered over the muscle belly of the anterior deltoid, equidistant from the clavicle and the insertion on the humerus [34]. The NIRS device automatically calculates the relative concentration of HbO_2_ in relation to the total amount of hemoglobin (tHb) (muscle oxygen saturation (SmO_2_) = HbO_2_/tHb)). During the bench press protocol, muscle oxygen saturation decreased during each set and returned to baseline levels during each 2 min rest period. To examine muscle oxygenation dynamics during the bench press sets and in recovery between sets, 4 variables were utilized. First, we calculated the loss of SmO_2_ (∆%SmO_2_) which is defined as the relationship between SmO_2_ at the beginning of each set (SmO_2_start) and SmO_2_ at the end of each set (SmO_2_stop). The SmO_2_start value was considered 1 s before each bench press set began, while the SmO_2_stop value was determined when the participant finished the concentric phase of the last repetition of each bench press set. The ∆%SmO_2_ variable was then calculated with the following formula derived from [35]:

$$\Delta {\%\mathrm{SmO}}_{2}=\left(\left(\frac{{\mathrm{SmO}}_{2}\mathrm{stop}\;\times 100}{{\mathrm{SmO}}_{2}\mathrm{start}}\right)-100\right)\times -1$$

Second, muscle reoxygenation time (SmO_2_RecT) was calculated as the amount of time to recover the muscle oxygenation following the final repetition of each bench press set [35]. That is, the time until the muscle oxygen saturation reached a value that stagnated for at least 5 s. Third, muscle oxygen resaturation rate (SmO_2_RecSlope) was determined which corresponds to the slope of the SmO_2_ signal for 30 s immediately following the final repetition of each bench press set [36]. Finally, we recorded and analyzed the highest SmO_2_ value achieved during each recovery period between sets (SmO_2_Peak).

#### 2.5.4. Stroop Test 

To evaluate executive function and mental inhibition, a Stroop task was administered using a web-based application on a standard laptop (https://www.psytoolkit.org/experiment-library/experiment_stroop.html; accessed on 12 April 2021) During the task, a black screen would display color names such as “yellow” in a different on-screen color. Participants were instructed to press a button on the keyboard that corresponded to the color displayed on screen, as soon as the word emerged with a maximum time limit of 2 s. Late responses were counted as an error. The Stroop task lasted for ~5 min and 3 variables are provided in the results (congruence, incongruence, and Stroop effect). Congruence is defined as the response time when color of the word and the meaning is the same (e.g., word “GREEN” is green in color). Incongruence is defined as the response time when the color of the word and the meaning is different (e.g., the word “GREEN” is red in color). The Stroop effect is defined as average response time in incongruent trials minus congruent trials [37]. Participants completed the Stroop task 3 separate times during each experimental trial visit: baseline, at the 30 min post ingestion, and following the muscular fatigue bench press exercise. The Stroop test is a well-documented prefrontal activation task indicative of components of executive function [37]. The Stroop test has previously been utilized to examine executive function following exercise and administration of dietary nitrates [9,15].

#### 2.5.5. Subjective Feelings and Ratings of Perceived Exertion 

Questionnaires were provided at baseline, 30 min post ingestion, and immediately after completion of the muscular fatigue bench press exercise. Participants were instructed to assess their subjective feelings of focus, energy, and fatigue using a 15 cm VAS. The scale was anchored by the words “low” and “high” to represent extreme ratings where the greater measured value represents the greater feeling. Questions were structured as “My level of focus is.” Participants were asked to rate their feelings at each time point by marking on the corresponding line. The validity and reliability of VAS have been previously established [38]. Following each experimental trial, participants were asked to provide a rating of perceived exertion (RPE) using the OMNI weightlifting scale [39] to indicate how difficult the workout was. The scale is from 0 to 10, with 0 being “extremely easy” and 10 being “extremely hard.”

### 2.6. Statistical Analysis

Before statistical procedures, all data were assessed for normal distribution, homogeneity of variance, and sphericity. If the assumption of sphericity was violated, a Greenhouse–Geisser correction was applied. A 2 (condition) × 4 or 5 (time point) repeated measures analysis of variance (ANOVA) was used to determine the effect of the supplement during each set on repetitions performed, all power measures, and all measures of muscle oxygenation. In addition, a separate 2 (condition) × 3 (time point) repeated measures ANOVA was used to determine the effect of the supplement on subjective feelings, cardiovascular measures, and the Stroop assessment. In the event of a significant F ratio, separate 1-way repeated measures ANOVA with Bonferroni adjustment was performed to assess the main effect for time during each condition, whereas separate dependent t-tests were used to assess conditional differences during each set. Dependent t-tests were used to determine differences in resting HR, BP, total repetitions, and OMNI scale RPE between treatments. For effect size, partial eta squared statistics were calculated, and according to [40] 0.01, 0.06, and 0.14 were interpreted as small, medium, and large effect sizes, respectively. Significance was accepted at an alpha level of *p* < 0.05, and all data are reported as mean ± SD.

## 3. Results

Nineteen participants were originally recruited for this investigation, of which, 4 participants had to drop out of the study due to scheduling conflicts. Additionally, 5 participants were not able to finish their second exercise condition due to COVID-19 virus gathering restrictions. 

### 3.1. Performance Measures 

**Figure 1:**
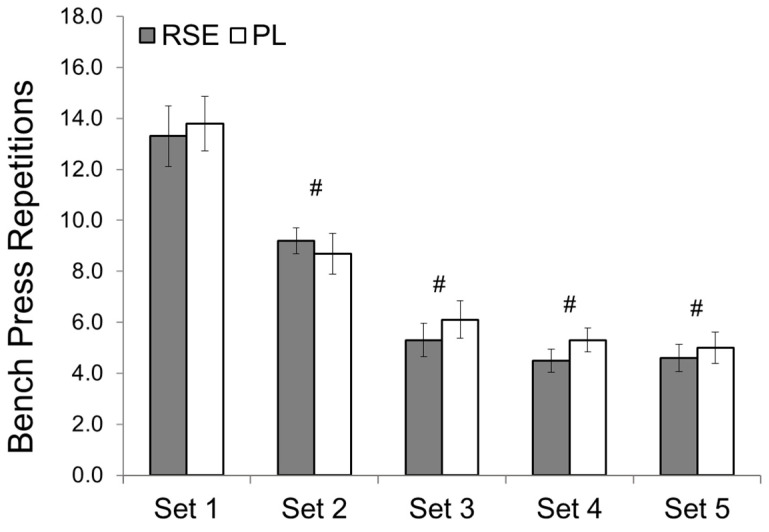
Number of repetitions completed during the barbell bench press exercise protocol at 75% 1-repetition maximum (1RM). RSE = Red Spinach Extract; PL = Placebo; # Significantly different (*p* < 0.05) than Set 1. Data presented as mean ± SD.

**Figure 2:**
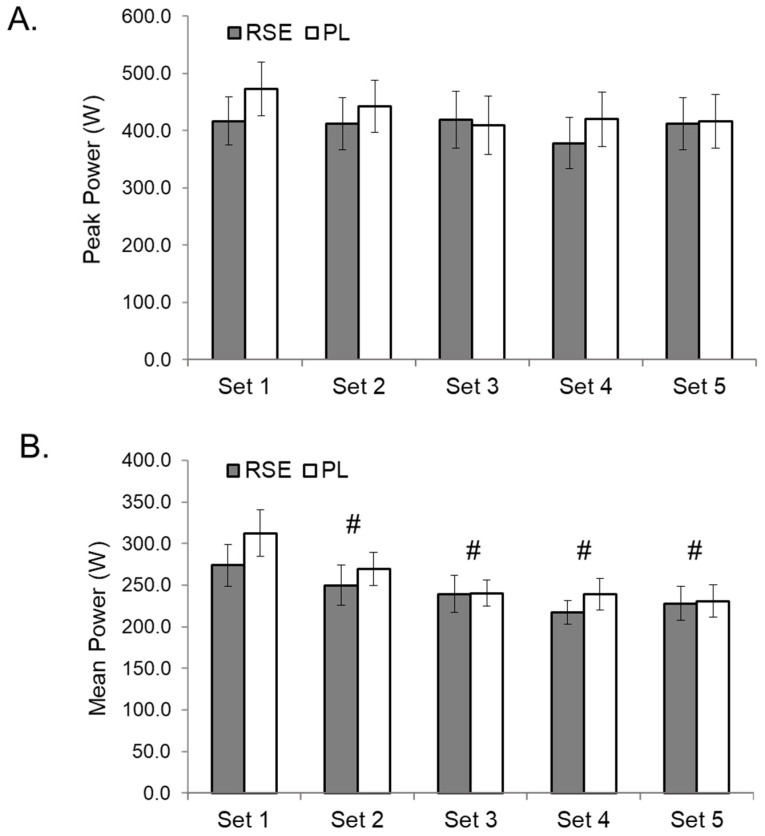
Changes in peak (**A**) and mean (**B**) power during the barbell bench press exercise protocol at 75% 1-repetition maximum (1RM). RSE = Red Spinach Extract; PL = Placebo; # Significantly different (*p* < 0.05) than Set 1. Data presented as mean ± SD.

There was no time by group interaction for bench press repetitions (*p* = 0.549, η^2^ = 0.034) (Figure 1). However, there was a main effect for time (*p* < 0.000, η^2^ = 0.872) with participants completing a significantly lower number of repetitions in sets 2–5 when compared to set 1 (*p* < 0.001). A paired t-test revealed no significant difference (*p* = 0.219) in total repetitions performed between treatments. There was no time by group interaction (*p* = 0.061, η^2^ = 0.061) or main effect for time for peak power (*p* = 0.076, η^2^ = 0.138) (Figure 2). There was no time by group interaction for mean power (*p* = 0.877, η^2^ = 0.029,). However, there was a main effect for time (*p* < 0.001, η^2^ = 0.021) with mean power significantly decreased in sets 2–5 (*p* < 0.001) compared to the first set. 

### 3.2. Muscle Oxygenation

There were no significant time by group interactions for ∆%SmO_2_ (*p* = 0.143, η^2^ = 0.095), SmO_2_RecT (*p* = 0.368, η^2^ = 0.058), SmO_2_RecSlope (*p* = 0.719, η^2^ = 0.026), or SmO_2_Peak (*p* = 0.713, η^2^ = 0.026) indicating similar responses between treatments (Table 1). A trend for a main effect for time was observed for SmO_2_RecSlope (*p* = 0.055, η^2^ = 0.137) with the recovery slope declining over the 4 rest periods between bench press sets. No other main effects for time were observed (*p* > 0.05).

### 3.3. Subjective Measures

There was no group by time interaction for subjective feelings of focus (*p* = 0.678, η^2^ = 0.021), energy (*p* = 0.600, η^2^ = 0.028), fatigue (*p* = 0.824, η^2^ = 0.010), and muscle pump (*p* = 0.867, η^2^ = 0.008) (Table 2). However, a main effect for time (*p* < 0.05) was found with significant increases in focus (*p* = 0.044) and muscle pump observed (*p* < 0.001) from baseline to pre-exercise. Additionally, ratings of focus (*p* = 0.004), fatigue (*p* < 0.001), and muscle pump (*p* < 0.001) were all significantly elevated at post-exercise compared to baseline measures. No significant differences in OMNI scale RPE measures were found between treatments (*p* = 0.468).

### 3.4. Stroop Test

There was no time by group interaction for congruence (*p* = 0.636, η^2^ = 0.301); however, there was a main effect for time (*p* = 0.001, η^2^ = 0.391) with congruence scores declining from baseline to 30 min (*p* = 0.008), and from test baseline to post-exercise (*p* = 0.001). There was no time by group interaction for incongruence (*p* = 0.110, η^2^ = 0.137). There was a main effect for time (*p* < 0.001, η^2^ = 0.512) observed with incongruence scores declining from 30 min to post-exercise (*p* < 0.01) and baseline to post-exercise to *(p* = 0.001). There was no time by group interaction for Stroop effect (*p* = 0.556, η^2^ = 0.014); additionally, there was no main effect for time (*p* = 0.698, η^2^ = 0.024).

### 3.5. Heart Rate and Blood Pressure

There was no time by group interaction for HR (*p* = 0.301, η^2^ = 0.064); however, there was a main effect for time (*p* < 0.001, η^2^ = 0.844) with HR declining from baseline to post-exercise (*p* < 0.001). There was no time by group interaction for systolic blood pressure (SBP; *p* = 0.717, η^2^ = 0.018); additionally, there was no main effect for time (*p* = 0.260; η^2^ = 0.072). There was no time by group interaction for diastolic blood pressure (DBP; *p* = 0.216; η^2^ = 0.082); additionally, there was no main effect for time (*p* = 0.948, η^2^ = 0.003).

## 4. Discussion

The scope of the current study was to determine if short term RSE supplementation (7 days), in addition to a single acute dose prior to exercise, enhances resistance exercise performance, muscle oxygenation, executive function, and subjective feelings of exertion in resistance-trained males. Our results indicate that supplementing with 2 g of RSE for 7 days has no significant effect on number of repetitions completed, peak power, and mean power during fatiguing upper-body resistance exercise. When compared to PL, RSE supplementation had no significant effect on any parameter of muscle oxygenation, cognitive performance, nor subjective feelings of focus, energy, and fatigue following the exercise bout. Furthermore, RSE supplementation did not alter HR, SBP, or DBP before or after exercise when compared to PL.

Our findings that RSE supplementation did not have a significant effect on resistance exercise performance are not in-line with recent studies regarding dietary NO_3_^−^ consumption and bench press performance [21]. Mosher and colleagues [21] reported that repetitions to failure and total weight lifted across 3 sets of the bench press at 60% 1RM was significantly greater following 6 days of NO_3_^−^ supplementation (400 mg) compared to a placebo treatment. Additionally, another study implemented a single dose of NO_3_^−^ (400 mg) 2 h prior to exercise and reported that acute BRJ supplementation significantly increased the total number of bench press repetitions completed across 3 sets to failure [22]. Furthermore, Williams et al. [22] also found that BRJ increased mean velocity and mean power output during the bench press across 2 sets of 2 reps at 70% of their 1RM when participants were instructed to perform the lift explosively. Thus, it appears that higher dosages of NO_3_^−^ have been more consistent in producing an ergogenic benefit for anaerobic exercise as improvements in peak isometric force production [24], high-intensity intermittent type exercise [18], and sport specific performance [41] have been observed following acute and chronic NO_3_^−^ doses of 800 mg or higher via BRJ. Conversely, 6 days of dietary nitrate loading (985 mg/day) showed no effect on countermovement jump performance, isometric strength, and muscular endurance in recreationally active males performing knee extension exercise [42]. Further, Trexler et al. [25] administered an acute 400 mg dose of NO_3_^−^ in the form of BRJ, 8 g of citrulline malate, and a placebo in a cross-over fashion to examine the effects of various NO precursors on muscular performance. Data from this study showed that neither citrulline malate nor BRJ significantly enhanced maximum leg extension performance. While data regarding anaerobic exercise and dietary NO_3_^−^ is equivocal, it is possible that our dosage of 180 mg of NO_3_^−^ was too low to observe an ergogenic benefit for resistance exercise similar to previous work [21,22]. However, recent work by Wylie et al. [43] demonstrates that skeletal muscle may serve as a NO_3_^−^ reservoir, suggesting that acute loading phases may increase NO3^−^ availability in the muscle during exercise. Consequently, we postulated that our aforementioned lower dose of NO_3_^−^ may be counteracted by the short-term loading phase. It has additionally been reported that NO_3_^−^ concentrations can vary significantly between different commercial products [44]. Thus, third party analysis of the actual NO_3_^−^ content of the RSE supplement along with plasma concentrations of NO_3_^−^ and NO_2_^−^ would have been helpful with interpreting our findings. 

Dietary nitrate increases NO concentrations which likely stimulates the production of cyclic guanosine (cGMP) by permitting the enzyme, soluble guanylate cyclase (sGC), to convert guanosine triphosphate (GTP) into cGMP [45]. In turn, cGMP initiates a signaling cascade resulting in relaxation of smooth muscle cells, inducing vasodilation, subsequently improving blood flow [45]. Ferguson et al. [19] demonstrated that dietary NO_3_^−^ improved skeletal muscle blood flow with greater perfusion observed in type II muscle fibers in a murine model. In the present study, NIRS derived estimates of muscle blood flow to the working muscles showed no difference between the RSE and PL treatments at any time point during the experimental visits. This coincides with data indicating no effect of RSE on flow-mediated dilation (FMD) [27]. Additionally, BRJ did not improve NIRS derived estimates of skeletal muscle oxygenation following upper-body ergometer [46], dynamic knee extensor protocol [47], or submaximal knee extensions in young males [25]. These findings differ from Bailey et al. [48] which demonstrated improved muscle oxyhemoglobin concentrations following BRJ consumption when cycling at higher exercise intensities which has also been observed in sustained isometric contractions [49]. Other investigations have shown improvements in upper-limb blood flow following chronic [50] or acute [1] consumption of BRJ. As data regarding improvements in limb blood flow is unclear, it would be helpful for prospective studies to determine the extent to which improved blood flow contributes to the performance benefits of dietary NO_3_^−^ on resistance exercise.

It has been demonstrated that acute and chronic dietary NO_3_^−^ consumption increases NO availability in the body, thereby reducing blood pressure via relaxation of smooth muscle [51]. The findings from our study suggest that 7 days of RSE supplementation had no effect on heart rate, SBP, or DBP when compared to PL. In accordance with these data, Haun et al. [27] did not find any differences in HR and BP at baseline or 30 min following 1 g of RSE consumption. Further, acute BRJ administration (140 mL) did not significantly affect HR, SBP, or DBP when consumed 2.5 h prior to a repeated sprint protocol [24]. Disagreeing with our findings, Gonzalez and colleagues [8] showed that 7 days of RSE supplementation, in addition to consuming the supplement 60 min pre exercise, appeared to lower DBP after a 4 km cycling time trial in recreationally active individuals. A possible explanation for the different BP responses may be due to the different types of exercise utilized for the testing and further studies should seek to compare the hemodynamic effects of dietary NO_3_^−^ between exercise modalities.

Wightman and colleagues [15] previously demonstrated that acute NO_3_^−^ supplementation significantly improved cerebral blood flow resulting in augmented cognitive performance assessed via a serial subtraction test in young healthy adults. To assess the impact of RSE on cognitive ability in the present study, we administered the Stroop test to our participants to assess executive function before and after a fatiguing bout of resistance exercise. When comparing RSE and PL, no significant differences for congruence, incongruence and total Stroop effect were noted. This corresponded with our data signifying RSE did not alter subjective measures of focus, energy, and fatigue via VAS which coincides with Mosher et al. [21] who also did not find a significant effect of BRJ on RPE following the bench press. Our findings partially support follow up work by this group which found BRJ supplementation only improved Stroop performance at baseline, finding no benefit during or following a yo-yo intermittent test [9]. Our data is in contrast to Thompson et al. [15] which found NO_3_^−^ consumption improved decision making reaction time without altering the accuracy of response during an intermittent sprint-cycling protocol designed to mimic the metabolic demand of a team sport. Interestingly, our data indicated that the fatiguing bench press protocol did not significantly alter the total Stroop effect when groups were collapsed. Thus, to achieve a cognitive ergogenic benefit from NO_3_^−^ consumption, the exercise stimulus may need to surpass a specific intensity or duration threshold. 

## 5. Conclusions

Our data suggests that 7 days of 2 g of RSE supplementation in addition to an acute dose did not have a significant effect on muscular fatiguing bench press exercise, cardiovascular measures, muscle oxygenation, and cognitive performance via the Stroop effect. Future NO_3_^−^ dosing studies are needed in regard to resistance exercise to determine the ergogenic threshold for this exercise modality. Additionally, as BRJ seems to provide more consistent improvements in resistance exercise performance thus far [21], a study comparing equal doses of BRJ and other forms of dietary NO_3_^−^ would help to determine if BRJ has unique benefits. For practical application, consumers should supplement with at least 400 mg of NO_3_^−^ to increase the likelihood of achieving an ergogenic benefit in resistance exercise.

## Figures and Tables

**Table 1:** Muscle oxygenation data during the bench press exercise and rest periods.

Variable		Set 1	Set 2	Set 3	Set 4	Set 5
**Bench Press Exercise**						
∆%SmO_2_	RSE	63.2 ± 19.7	69.4 ± 15.8	59.7 ± 16.1	61.5 ± 5.3	62.0 ± 21.3
	PL	69.0 ± 12.6	61.4 ± 15.8	60.7 ± 12.4	61.3 ± 17.1	66.5 ± 12.3
**Rest Periods**						
SmO_2_RecT (s)	RSE	60.8 ± 27.3	55.6 ± 18.8	53.2 ± 58.7	52.6 ± 17.4	-
	PL	57.8 ± 9.3	60.2 ± 14.0	58.7 ± 9.7	60.9 ± 12.1	-
SmO_2_RecSlope	RSE	1.14 ± 0.33	1.17 ± 0.41	0.91 ± 0.56	1.06 ± 0.68	-
	PL	1.26 ± 0.44	1.07 ± 0.32	0.86 ± 0.80	0.92 ± 0.49	-
SmO_2_Peak (%)	RSE	85.3 ± 3.43	86.1 ± 4.89	84.1 ± 5.28	84.6 ± 6.38	-
	PL	87.0 ± 3.24	87.3 ± 3.57	86.8 ± 3.60	86.1 ± 4.76	

Data presented as mean ± SD. RSE = Red spinach extract; PL = Placebo; SmO_2_ = skeletal muscle oxygenation; ∆%SmO_2_ = loss of muscle oxygenation; SmO_2_RecT = muscle reoxygenation time; SmO_2_RecSlope = muscle oxygen resaturation rate; SmO_2_Peak = highest SmO_2_ value achieved in rest period.

**Table 2:** Subjective measures and Stroop test data across to treatment sessions.

Variable		Baseline	PRE	IP
**Subjective Measures**				
Focus (cm)	RSE	8.3 ± 2.5	9.0 ± 2.7 #	9.8 ± 2.8 #
	PL	7.6 ± 2.6	8.7 ± 2.7 #	9.9 ± 1.8 #
Energy (cm)	RSE	7.7 ± 2.7	8.8 ± 2.3	7.4 ± 2.5
	PL	7.2 ± 2.9	8.5 ± 2.6	8.1 ± 2.7
Fatigue (cm)	RSE	4.7 ± 3.2	5.0 ± 2.7	10.1 ± 2.4 #
	PL	4.4 ± 2.9	4.5 ± 2.3	8.9 ± 3.2 #
Muscle Pump (cm)	RSE	3.9 ± 3.1	6.2 ± 3.3 #	11.2 ± 2.2 #
	PL	4.3 ± 4.1	6.6 ± 3.1 #	11.0 ± 2.4 #
Rating of Perceived Exertion (AU)	RSE	-	-	8.3 ± 0.7
	PL	-	-	9.9 ± 1.8
**Stroop Test**			
Congruence (ms)	RSE	720.1 ± 129.4	625.0 ± 72.9 #	607.3 ± 83.1 #
	PL	751.2 ± 148.0	648.6 ± 96.7 #	614.9 ± 90.8 #
Incongruence (ms)	RSE	831.9 ± 106.1	749.0 ± 92.2 #	740.9 ± 113.4 # †
	PL	877.4 ± 105.5	754.8 ± 91.3 #	772.5 ± 111.0 # †
Stroop Effect (ms)	RSE	111.8 ± 107.1	126.0 ± 64.5	135.6 ± 113.8
	PL	113.6 ± 72.4	106.1 ± 95.4	132.0 ± 54.5

Data presented as mean ± SD. RSE = Red spinach extract; PL = Placebo; PRE = Immediately prior to exercise; IP= Immediately post-exercise; # main effect of time compared to Baseline; † main effect for time compared to PRE.

## Data Availability

Data available upon reasonable request.
